# A Marvelous Story of Inoculated Croup

**Published:** 1877-03

**Authors:** 


A MARVELOUS STORY OF INOCULATED CROUP.—The *British Medical Journal* contains in a letter from Paris an account of the death of Dr. Regnault, from membranous croup, contracted by inoculation. The statement runs as follows: "Dr. Regnault had been attending a child suffering from so-called croup, and on November 25th, in performing tracheotomy, he accidentally inoculated himself with the virus through an abrasion of the skin on the finger. On the following day he went about as usual paying his morning visits, when, about noon, he was suddenly seized with giddiness and violent pains in the head, which were followed by febrile symptoms. A physician, a friend of the deceased, was immediately summoned to see him. This gentleman diagnosed 'angine couenneuse' (membranous or diphtheritic sore throat), which was confirmed by five other physicians who were called in consultation; and notwithstanding their assiduous attendance and persevering efforts to avert a fatal issue, the patient succumbed."

We are somewhat surprised that the editor of the *British*

*Medical Journal* should give currency to such a story. That a well developed case of diphtheria should be established in twenty-four hours, from the supposed inoculation, is an absurdity on its face, even admitting the possibility of propagating the disease by that means. We could never bring our mind to believe that Valleix and his medical friend, who both died in forty-eight hours after receiving in the throat a particle of supposed poison coughed up by diphtheritic patients, contracted the disease through that accident. So brief an incubation is inconsistent with all analogy. It has no parallel in pathology. Besides, though repeated experiments have been made by inoculation with diphtheritic material, and by applying it directly to the throat, as in the cases of Trousseau and Peter, in no one instance have the experimenters succeeded in communicating the disease by inoculation or contact. It is much more rational to presume in regard to Valleix and his friend—the only instances of the kind, we believe, on record—that they had already contracted the disease, and that the susception of the matter ejected was simply coincident.—*Pacific Medical and Surgical Journal.*

